# Weaning Stress Perturbs Gut Microbiome and Its Metabolic Profile in Piglets

**DOI:** 10.1038/s41598-018-33649-8

**Published:** 2018-12-24

**Authors:** Yuan Li, Yong Guo, Zhengshun Wen, Xuemei Jiang, Xin Ma, Xinyan Han

**Affiliations:** 1 The Key Laboratory of Molecular Animal Nutrition, Ministry of Education. Key Laboratory of Animal Nutrition and Feed Science in East China, Ministry of Agriculture, College of Animal Science, Zhejiang University, Hangzhou, 310058 China; 2 Institute of Animal Husbandry and Veterinary Science, Zhejiang Academy of Agricultural Sciences, Hangzhou, 310021 China; 3 School of Food Science and Pharmaceutics, Zhejiang Ocean University, Zhoushan, 316022 China

## Abstract

Weaned piglets are vulnerable to nutritional, physiological, and psychological stressors, leading to abrupt taxonomic and functional shifts in the intestinal microbiome. In this study, an integrated approach combination of 16S rDNA gene sequencing and the mass spectrometry-based metabolomics techniques was used to investigate the effects of weaning stress on intestinal microbial composition and its metabolic profiles of piglets. Three litters of suckling piglets with same parity were chosen. The samples of colonic contents were collected from each selected piglets (weaned day, 3 days after weaned) for microbial and metabolomics analysis. The results showed that *Lachnospiraceae*, *Negativicutes*, *Selenomonadales*, *Campylobacterales* and other 15 species increased after weaning, while *Porphyromonadaceace*, *Alloprevotella*, *Barnesiella* and *Oscillibacte*r decreased. Based on the function profiles prediction and metabolomic analysis, five key metabolic pathways including Phenylalanine metabolism, Citrate cycle (TCA cycle), Glycolysis or Gluconeogenesis, Propanoate metabolism, Nicotinate and nicotinamide metabolism might be the relevant pathways involved in weaning stress-induced gut microbiota dysbiosis. Taken together, these results indicated that weaning stress not only changed microbial composition and function but altered the microbial metabolic profiles in the intestine, which might provide a new insight in alleviating weaning stress and facilitating disease prevention during the period of weaning in piglets.

## Introduction

Enormous numbers and diverse microbiota population inhabit the gastrointestinal tract in human beings and animals, being responsible for gut maturation, pathogens resistance and immune modulation. Shaped by co-evolution more than millions of years, a symbiotic relationship has formed between gut microbiota and host. Gut microbiota play a critical role in nutrient metabolism and in return occupy the nutrient-rich environment^1^. Increasing evidence showed that the dysbiosis of gut microbiota was intimately associated with many diseases, including inflammatory bowel disease, cardiovascular diseases, allergies, diabetes and obesity^2,3^. Thus, improvement in host health requires a better understanding of intestinal environment especially the gut microbiota. Gut microbiota remains relatively stable once established. However, many factors such as host genetics, environment, diet, immunological pressures and antibiotics can cause dramatic changes in the microbial community^4^. Owing to the similarities to human beings in anatomy and nutritional physiology, pigs often served as a biomedical model of human^5,6^. Investigation of gut microbiota in pigs not only benefits to construct a healthy gut of the animal but also provide important evidence for human.

The pig gut microbiota shows dynamic composition and diversity which shifts over time and along the whole gastrointestinal tract^7^. Colonization was started at birth and shaped by consumption of the sow’s milk, building a milk-oriented microbiome^8^. Therefore, the suckling period offers a special window of gut microbiota modifications. Weaning is usually conducted at around 3–4 weeks in the modern swine industry, and piglets are fed with solid diets instead of liquid milk. It is a sudden, complex and highly stressful event in pig’s life. Weaning piglets are usually vulnerable to nutritional, physiological, and psychological stressors, leading to alterations of intestinal morphology, physiological function, and a shift in intestinal microbiome (e.g., increased potential pathogens and diarrhea)^9–11^. And the disruption of gut microbiota is regarded as one of the major factors leading to post-weaning diarrhea^12^. Previous study showed the temporal change of gut architecture and function after weaned could be divided two periods: an acute change (about 5 days) occurs immediately after weaned and an adaptive and maturity phase^13^. Thus 1–5days after weaned are considered to be a period in which piglets suffered the most severe weaning stress.

Recently, the association among gut microbiota, metabolites and host physiology has gained increasing attention. Microbial metabolites are indispensable to maintain the majority of the biological effects of gut microbiota. The extraction, synthesis and absorption of metabolites could be mediated by intestinal commensal bacteria^14^. Microbiota could exploit ingested dietary components, such as carbohydrates, proteins, and lipids to produce metabolites. These metabolites are mainly the short-chained fatty acids (SCFAs), as well as branched-chain fatty acids. Besides, there are some other metabolites produced by microbiota, including choline metabolites, bile acid metabolites, indole and phenolic derivatives. These metabolites have been appreciated for their beneficial effects on intestinal barrier, immune regulation and inflammation^15^. Therefore, it is of particular significance to probe the metabolic changes associated with weaning-perturbed gut microbiota. The present work was designed to investigate the effects of weaning stress on gut microbiome and its metabolite profiles in piglets via an integrated approach combining 16S rDNA gene sequencing and GC-TOF/MS (Gas Chromatography Tandem Time-of-Flight Mass Spectrometry) method.

## Results

### Gut Microbiota Diversity and Structure of Nursing and Weaned Piglets

**Figure 1:**
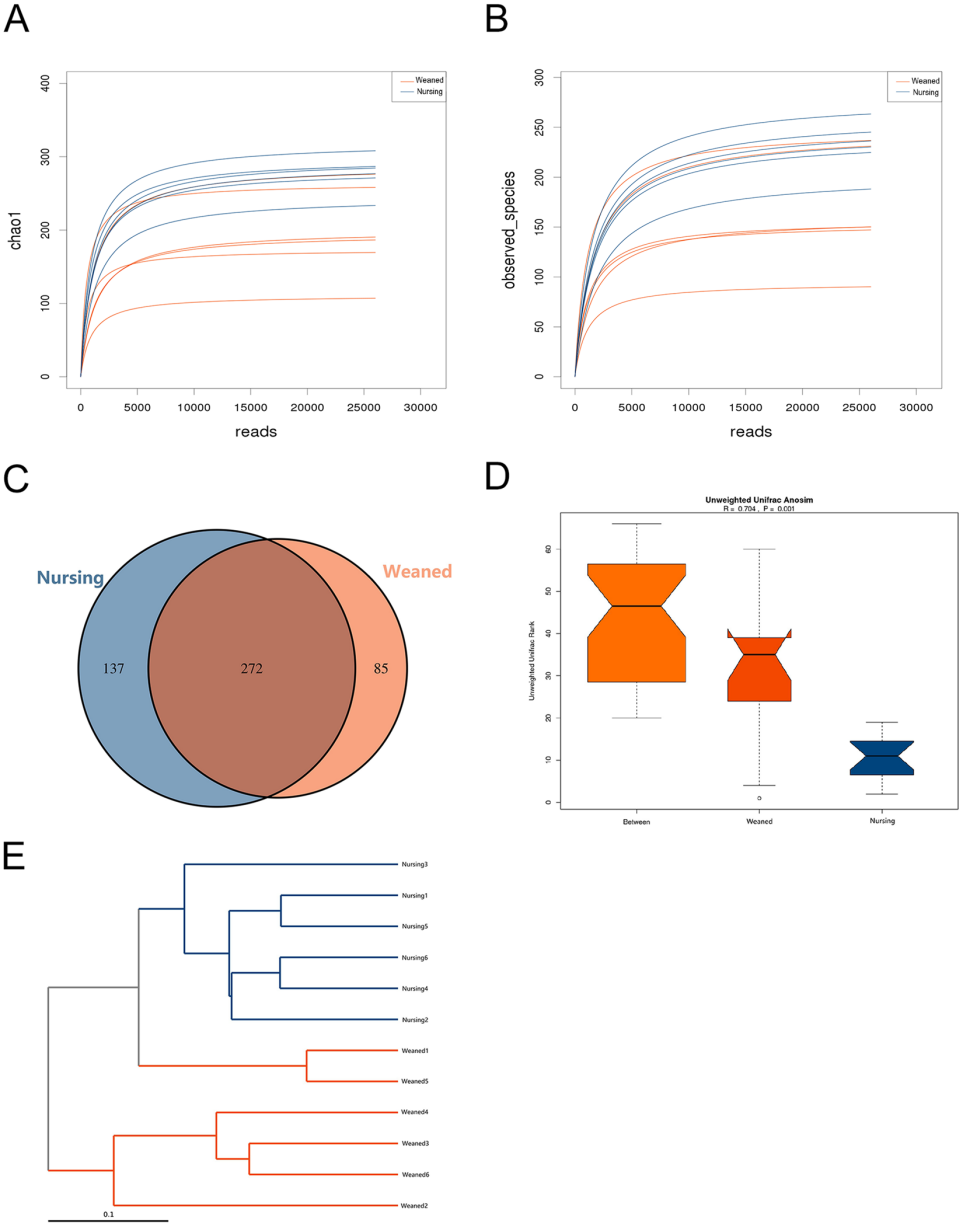
The overall structure of gut microbiota in nursing and weaned groups. (**A**), (**B**): Bacterial rarefaction curves based on Chao 1 and observed species index were used to assess the depth of coverage for each sample. (**C**) Venn diagram for bacterial OTUs compositions in two groups. (**D**) Similarity Analysis (Anosim Analysis) based on Unifrac algorithm. (**E**) Multiple sample similarity phylogenetic tree was built by Unweighted pair group method with arithmetic mean. Each sample was distinguished by different colors of lines. Blue and red represent nursing group and weaned group, respectively.

An average of 35595 high quality sequences per sample was obtained from 12 colonic content samples (Supplementary Table S1). The rarefaction Curves tended to be flat as shown in Fig. 1A,B, which demonstrated that almost all the bacterial species were detected in the colonic microbiota. Further, these sequences in the colonic contents of nursing and weaned piglets were assigned to 1,157 and 1,584 OTUs (operational taxonomic units) based on a 97% sequence similarity (Supplementary Table S2), respectively. Venn diagram reflected the number of OTUs that were common in groups as well as within the groups. This analysis showed there were 137 and 85 unique OTUs in weaned piglets and nursing piglets, respectively (Fig. 1C). The indexes of Chao1, observed species, PD-whole-tree, Shannon and Simpson were calculated to estimate alpha diversity. Shannon and Simpson indices, which reflect the species diversity, showed no significant difference in two groups. While, there were significant decrease in richness indices (Chao 1 and observed species) in weaned piglets compared to nursing piglets (Table 1). Unweifhted Unifrac Anosim and unweifhted Unifrac cluster tree based on UPGMA were used to analyze beta diversity (that is, diversity between individuals). Figure 1E showed a strong difference in the community composition of gut microbiota between two groups.

**Table 1:** Richness and diversity of indices of colonic microbiota in nursing and weaned groups.

Items	Richness indices		Diversity indices		
	Chao 1	Observed species	PD_whole_tree	shannon	simpson
nursing	306.86	255.33	22.2	5.24	0.94
weaned	223.06	185.33	15.33	4.76	0.91
p value	0.03	0.03	8.66E^−03^	0.24	0.24

The coverage percentage, richness estimators (Chao1, Observed species), and diversity indices (PD_whole_tree, Shannon and Simpson) were calculated using the QIIME program. PD_whole_tree: (phylogenetic distance) whole tree.

**Figure 2:**
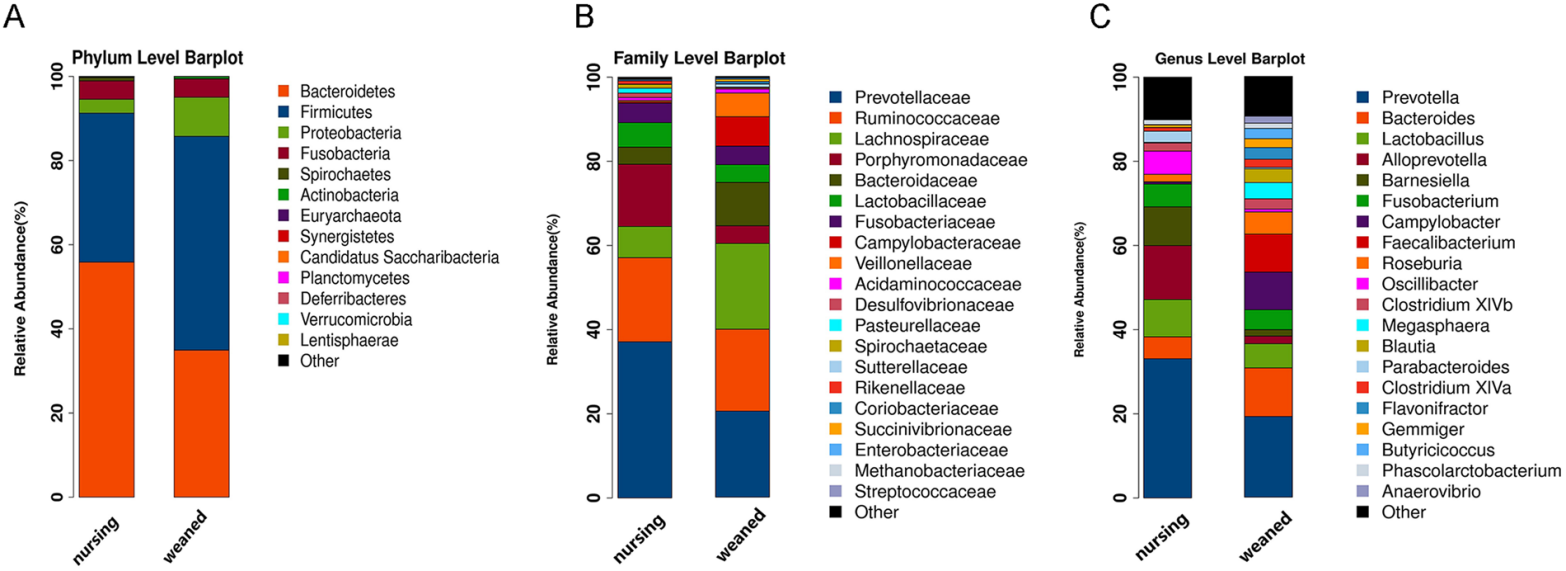
Gut microbial community structure in nursing and weaned groups. (**A**) Microbial community bar plot by phylum. (**B**) Microbial community bar plot by family. (**C**) Microbial community bar plot by genus. Each bar represents the average relative abundance of each bacterial taxon within a group. The top 20 abundant taxa are shown.

Four predominant phyla (*Bacteroidetes, Firmicutes*, *Proteobacteria, Fusobacteria*), which consisted of over 1% of total sequences on average, were found in colon as shown in Fig. 2A. There was no difference detected in dominant bacterial phyla between two groups (Supplementary Table S3). At family level, *Prevotellaceae* was predominant, followed by *Ruminococcaceae*, *Lachnospiraceae*, *Porphyromonadaceae*, and *Bacteroidaceae* (Fig. 2B and Supplementary Table S4). At genus level, *Precotella*, *Bacteroides*, *Lactobacillus* and *Alloprevotella* were the predominant genera (Fig. 2C and Supplementary Table S5).

### Differences in Microbiome between Nursing and Weaned Piglets

**Figure 3:**
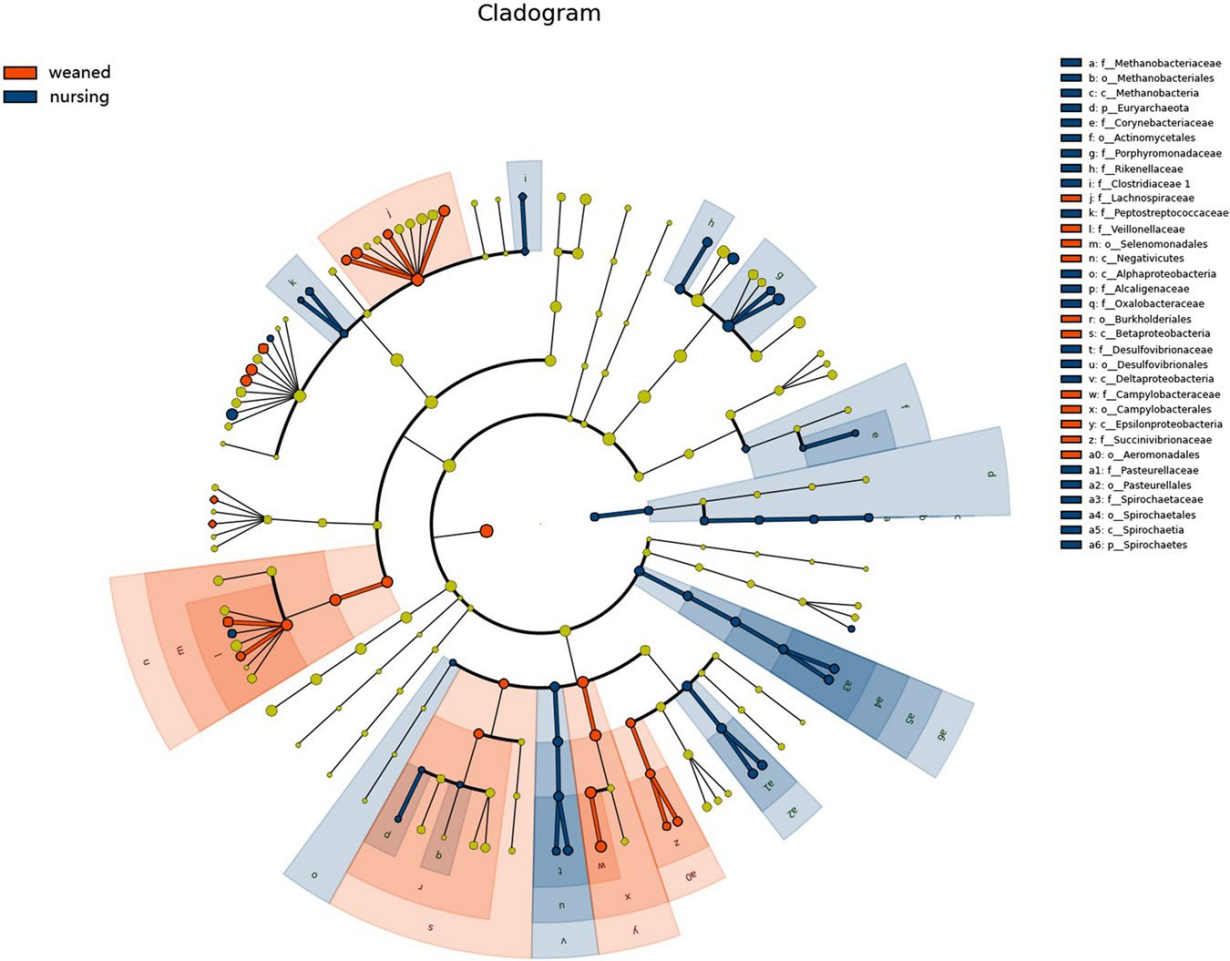
Cladogram. Bacterial taxa significantly differentiated between Nursing and Weaned group identified by linear discriminant analysis coupled with effect size (LEfSe) using the default parameters.

Figure 3 showed the microbiota with significant differences between nursing and weaned piglets. *Lachnospiraceae*, *Negativicutes*, *Selenomonadales*, *Campylobacterales* and other 15 species increased in weaned piglets, while *Porphyromonadaceace*, *Alloprevotella*, *Barnesiella* and *Oscillibacter* decreased.

### Predicted Function of Gut Microbiota in Nursing and Weaned Piglets

**Figure 4:**
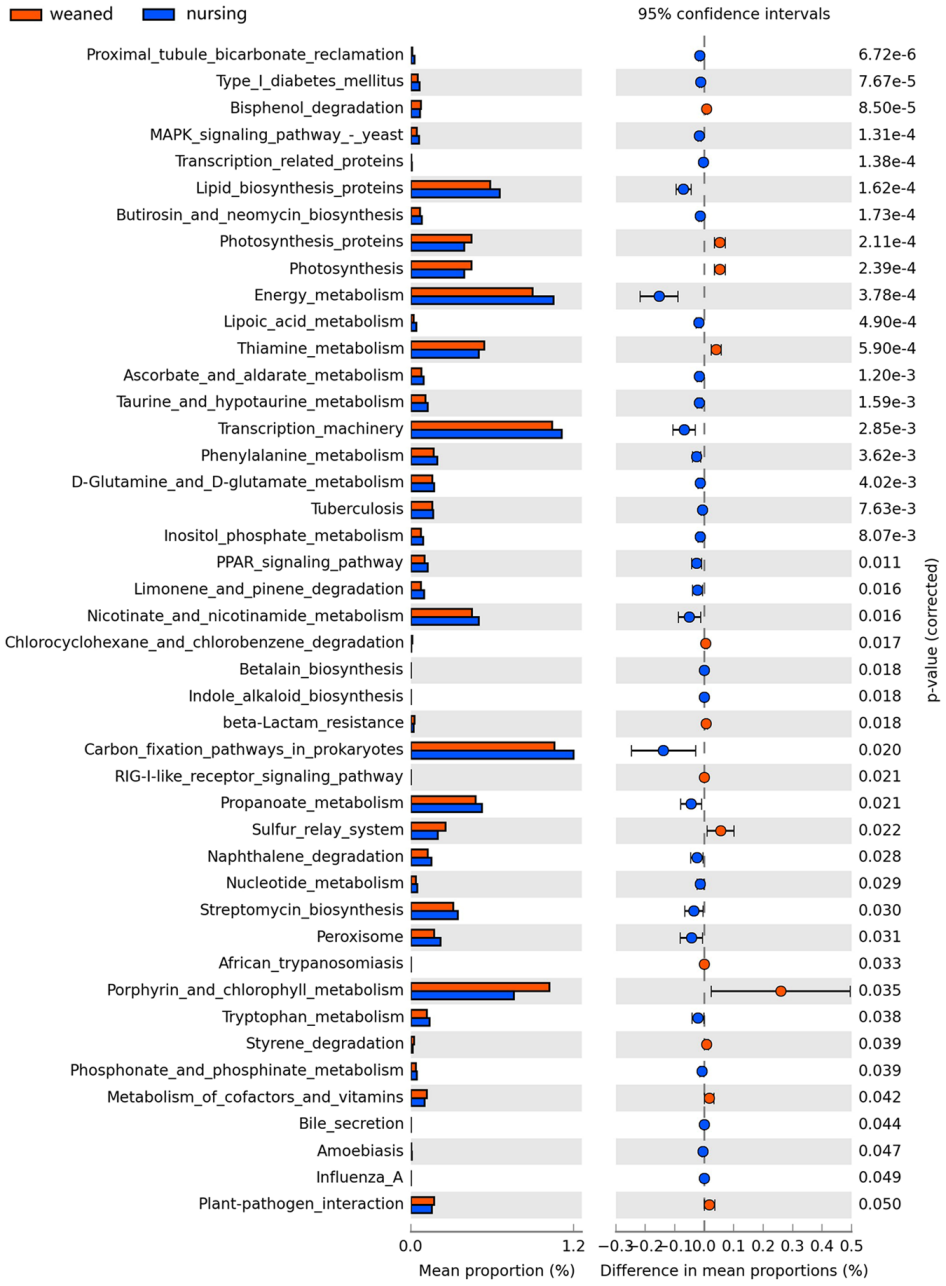
Predicted microbial function in the colon lumen of nursing and weaned groups. The third level of KEGG pathway was showed in the extended error bar. Blue and red represent nursing group and weaned group, respectively.

PICRUSt analysis was performed to predict the potential functions of gut microbiota. In comparison two groups, nursing piglets had higher enrichment of the pathways in Lipid biosynthesis proteins, Energy metabolism, Transcription machinery, Nicotinate and nicotinamide metabolism, Carbon fixation pathways in prokaryote, Propanoate metabolism, naphthalene degradation, Streptomycin biosynthesis and peroxisome. While other pathways such as Photosythesis proteins, Thiamine metabolism, Sulfur relay system were overrepresented in weaned piglets (Fig. 4).

### Metabolic Differences of Gut Microbiota between Nursing and Weaned Piglets

**Figure 5:**
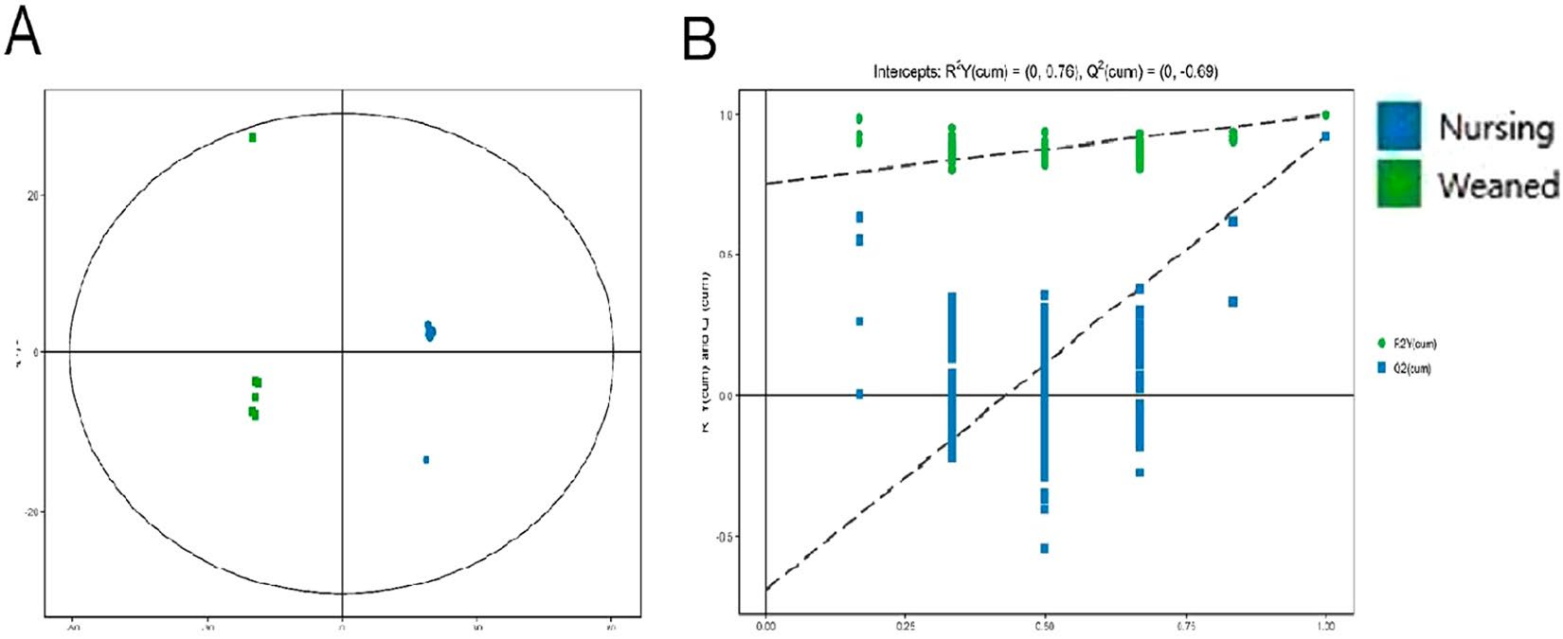
PCA 3D OPLS-DA score plots and corresponding validation plots of OPLS-DA derived from the GC-TOF/MS metabolite profiles in the colon lumen of nursing and weaned piglets. (**A**) OPLS-DA score plots showed significantly separated clusters in nursing and weaned group. (**B**) Permutation test of OPLS-DA model of nursing and weaned group. Blue and red represent nursing group and weaned group, respectively.

**Figure 6:**
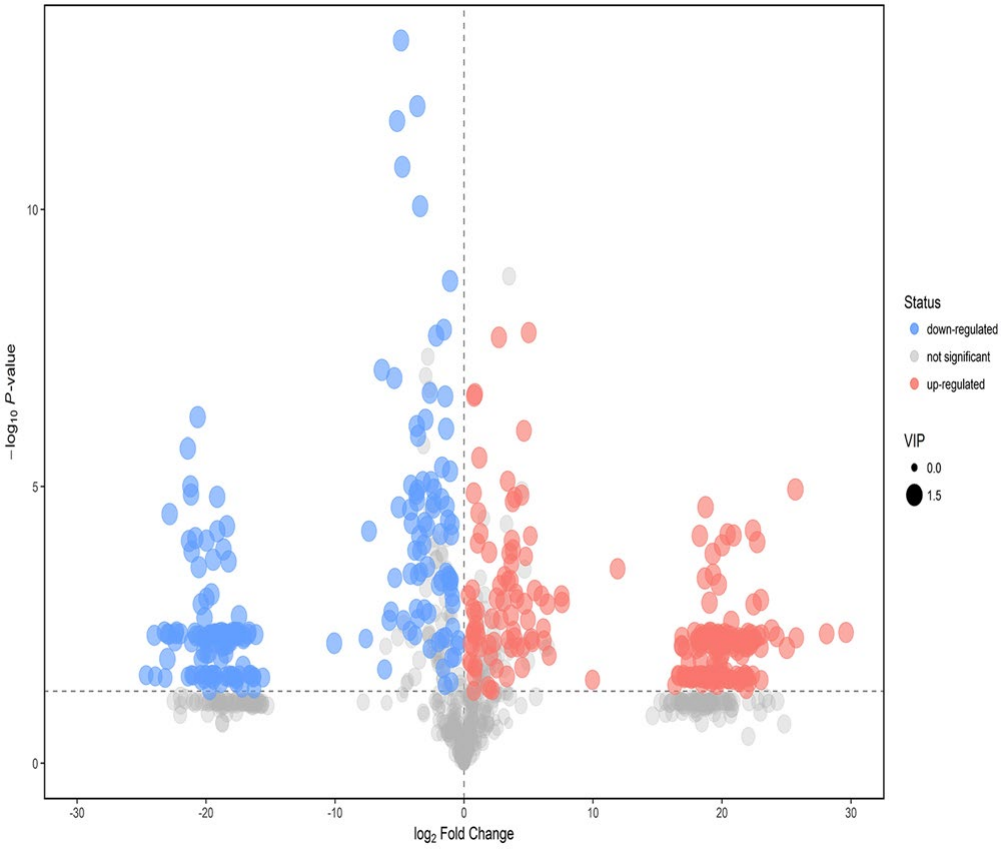
Volcano plot of nursing and weaned group. Each point represents a kind of metabolite, the scatter color represents the final screening result. Significant up-regulated metabolites are indicated in red, significantly down-regulated metabolites are indicated in blue, and non-significant differences in metabolites are grey.

Untargeted metabolomics analysis was performed to evaluate the metabolic differences of gut microbiota in two groups. An OPLS-DA method was performed to better understand the different metabolic patterns. Figure 5A showed significantly separated clusters between two groups. The values for R^2^X and Q^2^ and the results of permutation tests indicated that the samples were of reasonable quality (Fig. 5B). In total, relative levels of 433 biomarker metabolites differed significantly between nursing and weaned piglets. There was a clear distribution in metabolite between two groups (Supplementary Fig. 1). Figure 6 showed the variety of metabolic profiles in gut microbiome, with 239 increased and 194 decreased molecular features based on a VIP > 1 in 95% jack-knifed confidence intervals, respectively. And a total of 83 biomarker metabolites were filtered according to the standard with similarity >600, VIP > 1 and p < 0.05. These metabolites, including lipids, amino acids, carbohydrates, benzene, organic acids, amines and others, involved in multiple biochemical processes in the colon (Table 2).

**Table 2:** Identified metabolites for discriminating between nursing and weaned groups based on the untargeted metabolomics study.

Metabolic	Similarity	RT^a^ (min)	MEAN -Weaned	MEAN -Nursing	VIP	t-test^b^
**Lipids**						
heptadecanoic acid	828	20.337	5.57818E-11	0.000117	1.09225	*
Sphingosine	904	23.217	0.000373	0.004645	1.50233	***
phytosphingosine	872	24.471	0.000206	4.89435E-11	1.31316	**
alpha-Tocopherol	861	28.163	8.80993E-05	0.000391779	1.50347	***
Cholesterol	899	28.352	0.002760734	4.89435E-11	1.31090	**
Cholestan-3beta-ol	778	28.426	0.000120339	0.001103108	1.53157	***
Coprostan-3-one	767	28.529	5.57818E-11	8.6931E-05	1.54168	***
Stigmasterol	722	29.197	0.000277268	4.89435E-11	1.31269	**
L-Allothreonine	929	12.201	0.000465693	0.008418029	1.53677	***
2-Methylglutaric Acid	866	12.596	5.57818E-11	0.000126571	1.54227	***
Capric Acid	742	13.163	5.57818E-11	1.7529E-05	1.54125	***
lauric acid	708	15.517	0.001545153	0.002149401	1.11408	**
Myristic acid	850	17.694	0.000486967	0.040605731	1.54161	***
pentadecanoic acid	759	18.671	0.002088078	0.017616468	1.41982	**
palmitoleic acid	935	19.428	0.001754687	0.020718886	1.44486	***
stearic acid	893	21.495	0.040239752	4.89435E-11	1.30941	**
Arachidic acid	934	23.100	0.004225461	0.051756593	1.52418	***
Behenic acid	951	24.606	0.002671156	0.033936219	1.51484	***
Lignoceric acid	937	26.038	0.001859809	0.062046719	1.53010	***
Cerotinic acid	902	27.387	0.000111121	0.001223183	1.50911	***
**Carbohydrates**						
Lyxose	828	15.414	0.001186306	9.8097E-05	1.51917	***
xylose	953	15.589	0.031706877	0.002424436	1.51325	***
6-deoxy-D-glucose	807	16.220	0.000764127	0.000445482	1.34719	**
fructose	900	17.856	0.000335583	0.000993779	1.49148	***
Sedoheptulose	628	18.461	0.000268555	4.89435E-11	1.09879	*
D-galacturonic acid	864	18.579	0.000413546	4.89435E-11	1.31565	**
lactose	708	25.021	0.00031404	1.14153E-05	1.01325	***
trehalose	839	25.328	0.00033928	4.89435E-11	1.54237	***
maltose	775	25.296	0.000274583	0.000162243	1.03989	*
**Organic acids**						
Pyruvic acid	921	7.430	6.32753E-05	0.000116271	1.25376	**
glycolic acid	930	7.829	0.007297916	0.000592752	1.45508	**
3-Hydroxypropionic acid	843	8.826	0.00325326	8.7607E-05	1.39673	**
glutaric acid	708	12.458	0.001529942	0.000342019	1.28988	**
oxoproline	950	13.968	0.04241735	0.082504831	1.38890	***
**Amino acids**						
valine	810	8.041	0.001631261	0.000197131	1.46254	**
Maleimide	927	8.098	0.00013127	9.90848E-05	1.04632	**
sarcosine	733	8.732	5.97859E-05	0.000118821	1.08313	*
serine	907	10.480	5.57818E-11	0.000131568	1.54214	***
Isoleucine	841	10.923	0.000249148	8.0456E-06	1.35077	**
proline	960	11.050	0.002575346	0.012998171	1.50201	***
glycine	957	11.163	0.002625406	0.016532029	1.47505	***
beta-Alanine	945	12.773	0.001489759	9.94769E-05	1.43201	**
aspartic acid	956	13.927	0.00684775	0.015713239	1.25782	***
4-aminobutyric acid	844	14.067	0.007020642	0.000608468	1.51846	***
D-alanyl-D-alanine	755	14.881	0.000863753	0.000171924	1.33635	**
asparagine	865	15.681	5.57818E-11	0.000313104	1.30777	**
alpha-Aminoadipic acid	806	16.208	0.000124128	4.9774E-06	1.35393	***
glutamine	692	16.834	5.57818E-11	0.00042769	1.30790	**
ornithine	940	17.323	0.001909938	0.00740552	1.48481	***
citrulline	806	17.389	0.000271639	0.000529457	1.19390	*
histidine	833	18.369	5.57818E-11	0.00052798	1.09632	*
tyrosine	933	18.574	0.004176509	0.014777744	1.50781	***
**Benzene**						
m-Cresol	681	8.987	2.3733E-05	0.000171394	1.44746	***
Catechol	627	11.302	5.97445E-06	4.89435E-11	1.31768	**
phenylethylamine	627	11.302	5.97445E-06	4.89435E-11	1.31768	**
Phenyllactate	787	14.703	0.002295753	3.14665E-05	1.24267	**
gentisic acid	611	16.852	3.101E-05	4.89435E-11	1.54228	***
tyramine	846	18.329	0.014177717	4.89435E-11	1.30991	**
noradrenaline	705	20.732	8.42385E-05	4.89435E-11	1.54199	**
**Amines**						
Ethanolamine	882	10.600	0.001545522	0.00042396	1.35263	*
1,3-diaminopropane	928	15.449	0.001651697	0.000436322	1.25857	**
putrescine	920	16.513	0.163908181	0.02821584	1.50530	**
spermine	799	25.154	4.66359E-05	4.89435E-11	1.31451	**
**Organoheterocyclic compounds**						
2-Furoic Acid	604	8.695	2.60681E-05	4.89435E-11	1.54240	**
5-aminovaleric acid lactam	843	8.834	0.00394493	0.000152673	1.51907	**
nicotinic acid	976	11.021	0.000714674	0.001438858	1.34719	***
Orotic acid	635	16.509	5.57818E-11	7.6404E-05	1.08298	*
**Others**						
N-Methyl-DL-alanine	906	9.238	0.009567796	0.000546294	1.13615	**
Bis(2-hydroxypropyl)amine	622	13.296	5.57818E-11	3.53733E-05	1.28973	**
Digitoxose	727	15.012	5.57818E-11	5.96126E-05	1.09183	**
Cyclohexane-1,2-dione	793	15.685	2.64145E-05	4.89435E-11	1.11298	*
fucose	958	16.319	0.005013265	0.010654332	1.46744	***
Methyl-beta-D-galactopyranoside	650	17.678	0.000220863	0.001169638	1.46330	***
fructose	840	17.954	0.000134698	0.000284574	1.51000	***
Methyl Palmitoleate	740	18.258	4.88292E-05	0.000690577	1.16641	***
8-Aminocaprylic acid	734	18.511	0.002636825	4.89435E-11	1.54253	***
Gly-Pro	867	18.984	8.3268E-05	0.000492377	1.51841	***
xanthine	913	19.307	0.000738454	4.89435E-11	1.54224	**
N-Acetyl-D-galactosamine	920	19.958	0.000980973	0.001963837	1.39968	***
5-Methoxytryptamine	649	23.263	0.000471539	0.000281012	1.07002	**
DL-dihydrosphingosine	909	23.535	0.000341501	0.00271986	1.46487	***
adenosine	640	24.531	3.20881E-05	6.79893E-05	1.32913	***
Monoolein	662	25.420	2.73317E-05	7.12348E-06	1.48583	***

^a^Rentention time.

^b^Student t-test of the relative levels of compounds detected in weaned and nursing piglets(n = 6). *p < 0.05, **p < 0.01 and ***p < 0.001, respectively.

### Key metabolic pathways analysis

The commercial databases including KEGG (http://www.genome.jp/kegg/) and MetaboAnalyst (http://www.metaboanalyst.ca/) was applied to search for the pathways of metabolites. The result showed that beta-Alanine metabolism, Arginine and proline metabolism, Alanine, aspartate and glutamate metabolism, Nitrogen metabolism and other pathways were altered (Table 3). Among them, five metabolic pathways including Phenylalanine metabolism, Citrate cycle (TCA cycle), Glycolysis or gluconeogenesis, Propanoate metabolism and Nicotinate and nicotinamide metabolism were also found in the functional prediction of gut microbiota as shown in Fig. 4.

**Table 3:** Pathway analysis for nursing group and weaned group.

Pathway	Total^a^	Hits^b^	Raw p^c^	-ln(p)^d^	Holm adjust^e^	FDR^f^	Impact^g^
Beta-Alanine metabolism	17	6	0.0012	6.7056	0.0992	0.0992	0.6667
Arginine and proline metabolism	44	9	0.0055	5.2020	0.4404	0.1825	0.3289
Alanine, aspartate and glutamate metabolism	23	6	0.0068	4.9967	0.5340	0.1825	0.4633
Nitrogen metabolism	9	3	0.0277	3.5847	1	0.5618	0
Valine, leucine and isoleucine biosynthesis	11	3	0.0486	3.0246	1	0.6558	0.6667
D-Glutamine and D-glutamate metabolism	5	2	0.0520	2.9559	1	0.6558	0
Aminoacyl-tRNA biosynthesis	64	9	0.0567	2.8704	1	0.6558	0
Glycine, serine and threonine metabolism	32	5	0.0991	2.3117	1	0.9605	0.3528
Pantothenate and CoA biosynthesis	15	3	0.1067	2.2375	1	0.9605	0.0408
Phenylalanine metabolism	9	2	0.1526	1.8796	1	1	0.2222
Butanoate metabolism	20	3	0.2024	1.5974	1	1	0.0290
Ubiquinone and other terpenoid-quinone biosynthesis	3	1	0.2173	1.5265	1	1	0
Tyrosine metabolism	42	5	0.2280	1.4783	1	1	0.0532
Histidine metabolism	14	2	0.3012	1.1999	1	1	0.2662
Pyrimidine metabolism	37	4	0.3286	1.1129	1	1	0.0634
Pentose and glucuronate interconversions	15	2	0.3311	1.1052	1	1	0
Glutathione metabolism	26	3	0.3336	1.0978	1	1	0.0057
Cyanoamino acid metabolism	6	1	0.3877	0.9475	1	1	0
Biosynthesis of unsaturated fatty acids	42	4	0.4210	0.8651	1	1	0
Pentose phosphate pathway	19	2	0.4461	0.8072	1	1	0.1209
Propanoate metabolism	20	2	0.4732	0.7483	1	1	0
Citrate cycle (TCA cycle)	20	2	0.4732	0.7483	1	1	0.1398
Sphingolipid metabolism	21	2	0.4995	0.6942	1	1	0.0526
Methane metabolism	9	1	0.5213	0.6515	1	1	0
Vitamin B6 metabolism	9	1	0.5213	0.6515	1	1	0
Valine, leucine and isoleucine degradation	38	3	0.5838	0.5383	1	1	0
Riboflavin metabolism	11	1	0.5938	0.5212	1	1	0
Steroid hormone biosynthesis	67	5	0.6158	0.4849	1	1	0.0541
Nicotinate and nicotinamide metabolism	13	1	0.6555	0.4223	1	1	0
Primary bile acid biosynthesis	46	3	0.7143	0.3365	1	1	0.0667
Purine metabolism	68	4	0.7967	0.2274	1	1	0.0269
Lysine degradation	20	1	0.8068	0.2147	1	1	0
Pyruvate metabolism	22	1	0.8363	0.1788	1	1	0.1875
Starch and sucrose metabolism	23	1	0.8493	0.1633	1	1	0
Porphyrin and chlorophyll metabolism	25	1	0.8724	0.1365	1	1	0
Galactose metabolism	26	1	0.8826	0.1250	1	1	0.0161
Glycolysis or Gluconeogenesis	26	1	0.8826	0.1250	1	1	0.0989
Cysteine and methionine metabolism	28	1	0.9006	0.1047	1	1	0.0210
Glycerophospholipid metabolism	29	1	0.9085	0.0960	1	1	0
Steroid biosynthesis	35	1	0.9446	0.0570	1	1	0.0539
Amino sugar and nucleotide sugar metabolism	37	1	0.9532	0.0480	1	1	0
Fatty acid metabolism	39	1	0.9604	0.0404	1	1	0

^a^The number of metabolites in the pathway.

^b^The number of differential metabolites that hit the pathway.

^c^The P value of Metabolic pathway enrichment analysis.

^d^The P value of Multiple Hypothesis Test Corrected by Holm-Bonferroni Method.

^e^The P values of Multiple Hypothesis Tests for Corrected via the false discovery rate (FDR) Method.

^f^The Impact value of metabolic pathway topology analysis.

## Discussion

Weaning is a sudden, complex and highly stressful event in piglets’ life, accumulating evidence indicated that piglets have an abrupt taxonomic and functional shift in the intestinal microbiota after weaned^8,12^. However, the effects of weaning stress on microbial metabolites in piglets are still an unsolved issue. In this study, piglets at weaned day (nursing group) and 3 days after weaned (weaned group) were chose to investigate the effects of weaning stress on the composition of gut microbiota and its metabolites in the colon. The results showed that the diversity of gut microbiota in weaned piglets was similar with nursing piglets, while the richness decreased. These results were analogous with previous study, which found a continuously decreased alpha diversity until 11 days after weaned^16^. However, other studies presented opposite phenomenon. Those showed a continuous increase in alpha diversity of gut microbiota during weaning transition^17,18^. The reason might be that our studies focused on the gut microbiota during the early period after weaning, whereas their studies investigated the gut microbiota during the period from weaning to adulthood in pigs. It seems that alpha diversity of gut bacterial community undergoes decreases first during the early period after weaning, and then increases from weaning to adulthood in pigs on the whole.

In line with previous studies, *Bacteroidetes* and *Firmicutes* were the two most dominant phyla in the intestine of piglets, regardless of weaning^16,19^. *Proteobacteria* and *Fusobacteria* were the followed abundant phylum. There was no difference in bacterial dominant phyla in the colon between nursing and weaned piglets. And *Candidatus Saccharibacteria*, *Planctomycetes*, *Verrucomicrobia* and *Lentisphaerae* even cannot be detected in the gut of weaned piglets, suggesting that these bacterial species may not adapt to the intestinal tract environment after weaning. What’s more, the results showed that several bacterial species had a remarkable difference between nursing and weaned piglets. For example, *Alloprevotella* and *Oscillospira* were decreased, while *Campylobacterales*, *Campylobacteraceae* and *Campylobacter* were increased in weaned piglets. *Alloprevotella* mainly produce succinate and acetate, which could improve the gut barrier and exhibit anti-inflammatory function^20^. *Oscillospira* species are butyrate producers and can use host glycans as a source of energy^21^. Study found that the presence of *Oscillospira* is reduced in diseases that involve inflammation^22^. *Campylobacterales* was one of opportunistic pathogens, causing a life-threatening gastrointestinal disease^23^. And *Campylobacteraceae* and *Campylobacter* were also increased after weaning. The increase of this bacterial species in weaned piglets may be one of the major reasons of post-weaning diarrhea. These results indicated that unhealthy alterations in the composition of gut microbiota triggered by weaning stress.

Gut microbiota participate in many functional metabolic pathways through which nutrient digestion and absorption, lipid metabolism, and hormone biosynthes are influenced^24^. To investigate how the functional capacity of the gut microbiota changed after weaning in piglets, Functional metagenomics prediction approach was used to analyze the KEGG pathways compositions in microbial populations. The results showed that many metabolic pathways such as lipid biosynthesis proteins, energy metabolism and phenylalanine metabolism were changed during the period of weaning. Gut microbiota could also directly alter its metabolic capacity through microbial products, affecting intestinal function. Butyrate, a kind of metabolite, produced by gut microbiome could regulate energy metabolism and autophagy in the mammalian colon^25^. So GC-TOF/MS method was applied in the current study to quantitatively measure small molecular metabolites in biological samples and identify relevant Pathways. Based on PICRUSt and metabolomics analysis, we found that five metabolic pathways including Phenylalanine metabolism, Citrate cycle (TCA cycle), Glycolysis or Gluconeogenesis, Propanoate metabolism and Nicotinate and nicotinamide metabolism were the key relevant metabolic pathways involved in weaning stress-induced gut microbiota dysbiosis.

Previous study indicated that alterations in the microbiota composition elicited by nutrition are associated with neurotransmitter concentrations^26^. Nutrition can influence hormonal, neurotransmitter, and signalling pathways, which in turn modulate several brain processes impacting both appetite and mood^27^. Phenylethylamine, as a neuromodulator or neurotransmitter, participates in the phenylalanine metabolic pathway and plays an important role in mood regulation^28^. In the present study, the phenylethylamine level decreased in weaned piglets, which indicated piglets had a bad mood caused by stress. And a low level of phenylethylamine could not modulate the mood well, which may cause a decreased feed intake and weight gain of piglets. In addition, changed neurotransmitter noradrenaline and 4-aminobutyric acid level were also found in the present work. Gut microbiota could influence the immune system via neurotransmitters, and proinflammatory cytokines released through microbiota-induced immune response can in turn have a direct influence on the secretion of neurotransmitters such as norepinephrine^29^. Compelling studies have linked weaning to an upregulation of expression of inflammatory cytokines in the intestine of piglets^10,30^. Thus the regulatory mechanism and potential role of “microbiota-brain-gut” axis that play during weaning period in piglets are warranted to further investigation.

During a short period after weaning, small intestine length, microvilli height, crypt depth, and barrier function of piglets were changed. What’s more, digestive enzyme activity and absorptive capacity of small intestine were reduced, resulting in a low absorption capacity of amino acids and carbohydrates^31^. In the present study, decreased essential amino acids such as valine and some carbohydrate in weaned piglets supported this view. TCA cycle is responsible for the oxidative degradation of sugars, fats, and amino acids and could link different types of nutrients together. Pyruvic acid, as an important intermediate involved in the Citrate cycle (TCA cycle), was decreased in the weaned piglets. The reduction in energy metabolism may be part of the gut adaptation process. Microorganisms may engage energy for adaptation mechanisms to ensure necessary physiological functions^32^.

Notably, pathways related to short chain fatty acid (SCFA) metabolism such as propanoate metabolism were enrichment in piglets. Functional metagenomics prediction analysis using PICRUSt revealed that genes involved in Propanoate metabolism were more abundant in nursing piglets. And the bacteria *Oscillospira*, known to be as a butyrate producer, decreased in the gut microbiota of weaning piglets, which might indicate the decreased SCFA production after weaning. SCFAs are major anions in the gut and could be absorbed rapidly by colonic epithelial cells. Growing evidence proved that these small molecules could protect the host against colonic diseases, improve the gut barrier function and exhibit anti-inflammatory effects^33,34^. In addition, SCFA-mediated beneficial metabolic effects on health can be mediated by induction of intestinal gluconeogenesis^35^. A recent study showed that SCFAs could also improve the feed efficiency of swine^36^. Thus the reduction in Propanoate metabolism of weaned piglets in the current study might be related to a low growth performance and unhealthy gut environment after weaning.

Administration of niacin (nicotinic acid and nicotinamide) has been shown to beneficially effect the host-microbiome interaction in a mouse model^37^. Nicotinamide adenine dinucleotide (NAD+) is the central cofactor of metabolism, mediating ATP generation, reactive oxygen species (ROS) detoxification, biosynthetic processes, DNA repair, and nutritionally sensitive gene regulation^38^. In the present study, decreased nicotinic acid was found in weaning piglets, which indicated an adverse influence on piglets trigged by stress.

## Conclusion

In conclusion, our current work demonstrated that weaning stress not only disturbed the gut microbiota but also altered the metabolome profiles in the colon. After weaning, some benefit bacterial species such as *Alloprevotella* and *Oscillibacter* decreased in weaned piglets, while some opportunistic pathogens such as *Campylobacterales*, *Campylobacteraceae* and *Campylobacter* increased. In addition, based on PICRUSt and metabolomics analysis, five key metabolic pathways including Phenylalanine metabolism, Citrate cycle (TCA cycle), Glycolysis or Gluconeogenesis, Propanoate metabolism, Nicotinate and nicotinamide metabolism were identified as the relevant pathways associate with weaning stress-induced gut microbiota dysbiosis. These results may provide a new insight in alleviating weaning stress of piglets and facilitating disease prevention during weaning by manipulating host-microbiome metabolites interactions. And future study might focus on the potential functions of neurotransmitters in gut microbiome, neurogenesis, behavior and immune of piglets suffering weaning stress.

## Materials and Methods

### Animals and Sample Collection

All methods in this study were performed in accordance with the Guide for the Care and Use of Laboratory Animals prepared by the Institutional Animal Care and Use Committee of Zhejiang University, and animals used in this experiment were approved by the principles of the Zhejiang University Animal Care and Use Committee (NO. 2012-0178). Three litters of suckling piglets (21d, Duroc × Landrace × Yorkshire) with same parity were chosen from a commercial pig farm located in Ningbo city, Zhejiang province, China. Two barrows from each litter were randomly selected and humanely euthanized. The samples of colonic content were collected and transferred into sterile conical tubes. Then remaining piglets in the same trial were separated from sows and transferred into the same pen. These weaned piglets were fed a corn/soybean- based diet and had free access to water and feed. All nutrients reached or exceeded NRC (2012) recommendations for piglets. Three days after weaned, two barrows from each litter were selected and humanely euthanized. The samples of colonic content were collected into sterile conical tubes. All collected colonic samples were immediately frozen in liquid nitrogen and stored at −80 °C.

### DNA extraction and 16S rDNA gene amplicon sequencing

Total Microbial genomic DNA extraction was performed from colonic lumen using E.Z.N.A.® Stool DNA Kits (Omega Bio-tek, Norcross, US) according to the instructions of the manufacturer. The method of microbial community analysis was performed according to previous study^39^. Bacterial univers alprimers (341 F: ACTCCTACGGGAGGCAGCAG, 806 R: GGACTACHVGGGTWTCTAAT) were used for the amplification of the V3–V4 region of the bacterial 16S rDNA gene and subsequent pyrosequencing of the PCR products. Amplicons were confirmed by electrophoresis on a 2% agarose gel, purified with the AxyPrep DNA kit (AXYGEN, Tewksbury, MA, US), quantified by Qubit 2.0 Fluorometer (Thermo Fisher Scientific, Waltham, US) to pool into even concentration. Amplicon libraries were sequenced on Illumina Miseq PE250 platform (Illumina, San Diego, US).

### Sequencing data analysis

The paired-end reads were obtained 2 × 250 bp using MiSeq platform. In order to obtain high quality sequences, tags with length of <220 nt, average quality score of <20, and tags containing >3 ambiguous bases were removed by PANDAseq. Data was de-chimeric and clustered using Usearch software, a standard clustering of 97% similarity was applied to obtain OTU (Operational Taxonomic Units). And the taxonomy-based analysis to the OTUs was performed by RDP algorithm using GreenGene database (http://greengenes.lbl.gov). QIIME was used for analyzing alpha diversity, which included calculation of the observed species, Chao 1, Shannon, and Simpson indices. And principal coordinate analysis (PCoA), Anosim Analysis, and unweighted pair group method with arithmetic mean (UPGMA) were performed using QIIME for beta-diversity analysis. Linear discriminant analysis coupled with effect size (LEfSe) was applied to identify the bacterial taxa differentially represented between groups at genus or higher taxonomy levels^40^.

### Metagenome prediction based on 16S rDNA gene data

Phylogenetic Investigation of Communities by Reconstruction of Unobserved States (PICRUSt), which makes up for the shortage of 16S rDNA gene studies, was used for predicting the gene family abundances of bacterial populations based on the precalculated GreenGenes (v13.5) database. In brief, the analysis process is divided into two steps: gene content inference and metagenome inference. The software STAMP was conducted to detect the differentially abundant Kyoto Encyclopedia of Genes and Genomes (KEGG) pathways in two groups with false discovery rate correction.

The rank sum test was used to identify species with significant differences between groups. Two-sided Welch’s t-test and Benjamini-Hochberg FDR correction were performed in two-group functional prediction analysis. P < 0.05 was considered statistically significant. And the prediction accuracy of PICRUSt was evaluated by the Nearest Sequenced Taxon Index (NSTI), with lower value indicating a higher accuracy of prediction.

### Sample preparation for GC-TOF/MS analysis

Briefly, approximately 1.0 g colonic contents were mixed with 3 ml 4 °C H_2_O, followed by centrifugation at 3,200 × g for 15 minutes. 50 μl of the supernatant was taken and added with 200 μl of methanol, which contains 12.5 μg/ml of [13C2] myristic-acid. The mixture was maintained at 4°C for 1 h and subsequently centrifuged at 20,000 g for 10 minutes. Then the supernatant fraction was transferred to a glass vial and dried in a vacuum concentrator. And added methoxyamine salt reagent (methoxyamine hydrochloride, dissolved in pyridine 20 mg/mL), incubated in an oven at 80 °C for 30 min, then derivatized using N, O Bis (trimethylsilyl) trifluoroacetamide (BSTFA), incubated for 1 hour at 70 °C. The final mixture was strongly vortexed for 1 min and then subjected to detection.

### GC-TOF/MS analysis

The derivatized samples were analyzed using an Agilent 7890 GC system equipped with a Pegasus 4D TOFMS (LECO, St. Joseph, MI) with a DB-5MS capillary column (30 m × 250 μm inner diameter, 0.25 μm film thickness coated with 95% dimethylpolysiloxane cross-linked with 5% diphenyl under the following conditions: initial temperature was kept at 80 °C for 0.2 min, increased to 180 °C at a rate of 10 °C/min, to 240 °C at a rate of 5 °C/min, and further to 290 °C at a rate of 20 °C/min; the column was then maintained for 11 min. One μL of sample solution was injected with helium as the carrier gas at a flow rate of 1 mL/minute. The temperatures of transfer line, and ion source were 245 °C, and 220 °C, respectively. The MS data were acquired with a mass-to-charge ratio (m/z) range of 20−600 in a full-scan mode.

### GC-TOF/MS data acquisition and processing

The raw peaks extraction, data baselines filtering and calibration, peak alignment, deconvolution analysis, peak identification, and integration of the peak area were operated using the Chroma TOF4.3X software (LECO) and LECO-Fiehn Rtx5 database. The peak identification was tested using the RI (retention time index) method. The missing values of the original data were filled by half of the minimum value via a numerical simulation method. Noise removal was performed based on an interquartile range to filter data, then, data were normalized by area normalization methods. The SIMCA14.1 software package (Umetrics, Umea, Sweden) was used for multivariate variable pattern recognition analysis: principal component analysis (PCA), partial least squares discriminant analysis (PLS-DA), and orthogonal partial least-squares discriminant analysis (OPLS-DA). PCA was used to show the internal structure of the data and display the similarity and difference. OPLS-DA was applied to obtain a higher level of group separation and better explain the variables. To evaluate the predictive ability and fitting level of the model, the parameters R^2^Y and Q^2^ were applied. The metabolites responsible for differentiating two groups were filtered with the following requirements: variable importance in the projection (VIP) >1 and P-values of 0.05 (threshold) with 95% Hotelling’s T-squared ellipse.

Data was analyzed by SPSS 20.0 using Student’s t-test to profile metabolite differences between two groups. The commercial databases including KEGG (http://www.genome.jp/kegg/) and MetaboAnalyst (http://www.metaboanalyst.ca/) was utilized to search for the pathways of metabolites. We can further screen pathways and find the key pathways with the highest correlation with metabolites through comprehensive analysis of the pathways where the differential metabolites are located (including enrichment analysis and topological analysis).

## Electronic supplementary material

Supplementary Material

## Data Availability

All data generated or analysed during this study are included in this article and its Supplementary Information files.
