# Supplementary material for: Weaning Stress Perturbs Gut Microbiome and Its Metabolic Profile in Piglets
Source: Sci Rep. 2018 Dec 24;8:18068. doi: 10.1038/s41598-018-33649-8 (PMC6305375; doi:10.1038/s41598-018-33649-8)
Supplement: Supplementary file 1 — Supplementary Material [file 41598_2018_33649_MOESM1_ESM.docx]

***Supplementary Material***

Weaning Stress Perturbs Gut Microbiome and

Its Metabolic Profile in Piglets

Yuan Li^1^, Xuemei Jiang^1^, Xin Ma^1^, Saisai Cheng^1^, Shijie Geng^1^, Xinyan Han*^1^

*Corresponding author

Xinyan Han

College of Animal Science, Zhejiang University，866 Yuhangtang Road, Hangzhou 310058, China

Tel: +86-0571-88982446

Fax: +86-0571-88982650

E-mail: [xyhan@zju.edu.cn](mailto:xyhan@zju.edu.cn)

**Table S1** **The sequences of each sample in colonic contents** **of nursing and weaned piglets (n=6)**

| **Sample** | **Clean Reads^a^** | **Bases(bp)^b^** | **Q20(%)^c^** | **Q30(%)^d^** | **GC(%)^e^** | **Average length(bp)^f^** |
| --- | --- | --- | --- | --- | --- | --- |
| Weaned6 | 34729 | 14228621 | 95.68% | 92.62% | 53.21% | 409 |
| Weaned5 | 35573 | 14858091 | 94.71% | 91.23% | 50.99% | 417 |
| Weaned4 | 35260 | 14489685 | 95.63% | 92.52% | 53.07% | 410 |
| Weaned3 | 34666 | 14243111 | 95.58% | 92.47% | 53.37% | 410 |
| Weaned2 | 35138 | 14731095 | 94.92% | 91.62% | 50.36% | 419 |
| Weaned1 | 38994 | 16156088 | 95.25% | 92.04% | 52.08% | 414 |
| Nursing5 | 33749 | 14066316 | 94.70% | 91.15% | 53.73% | 416 |
| Nursing4 | 33994 | 14171670 | 95.09% | 91.74% | 53.73% | 416 |
| Nursing6 | 33401 | 13951973 | 94.69% | 91.17% | 52.99% | 417 |
| Nursing1 | 36594 | 15217278 | 95.04% | 91.68% | 53.74% | 415 |
| Nursing3 | 36178 | 15131736 | 94.98% | 91.59% | 52.32% | 418 |
| Nursing2 | 38865 | 16198744 | 94.99% | 91.64% | 53.76% | 416 |
| Mean reads | 35595 |  |  |  |  |  |

^a^ Clean Reads： the number of valid sequences

^b^ Bases (bp) ：the total number of bases

^c^ Q20 (%)：the percentage of Q20 bases that make up the total number of bases

^d^ Q30 (%)：the percentage of Q30 bases that make up the total number of bases

^e^ GC (%)：the GC content of the sample

^f^ Average length (bp) the average length of the sample

**Table S2** **The OTU numbers of each sample in colonic contents of nursing and weaned piglets (n=6)**

| **sample_name** | **clean reads** | **mapped reads** | **mapped ratio(%)** | **OTUs** |
| --- | --- | --- | --- | --- |
| Weaned1 | 38994 | 33771 | 86.60563164 | 265 |
| Weaned2 | 35138 | 31111 | 88.53947294 | 104 |
| Weaned3 | 34666 | 29659 | 85.55645301 | 174 |
| Weaned4 | 35260 | 32747 | 92.87294385 | 181 |
| Weaned5 | 35573 | 30875 | 86.79335451 | 264 |
| Weaned6 | 34729 | 29536 | 85.04707881 | 169 |
| Nursing1 | 36594 | 32563 | 88.98453298 | 266 |
| Total OTUs |  |  |  | 1157 |
| Nursing2 | 38865 | 32741 | 84.24289206 | 272 |
| Nursing3 | 36178 | 32127 | 88.80258721 | 220 |
| Nursing4 | 33994 | 28834 | 84.82085074 | 255 |
| Nursing5 | 33749 | 28813 | 85.37438146 | 296 |
| Nursing6 | 33401 | 29428 | 88.10514655 | 275 |
| Total OTUs |  |  |  | 1584 |

The OTUs were identified based on 97% sequence similarity**.**

**Table S3 The phyla composition of colonic microbiome in nursing and weaned piglets (n=6)**

| **tax_name** | **Weaned(n=6)** | **Nursing(n=6)** | **p** |
| --- | --- | --- | --- |
| *Bacteroidetes* | 34.92±24.72% | 55.85±1.83% | 0.07 |
| *Firmicutes* | 50.82±34.46% | 35.42±8.48% | 0.31 |
| *Proteobacteria* | 9.27±10.26% | 3.27±1.03% | 0.20 |
| *Fusobacteria* | 4.38±6.79% | 4.39±7.02% | 0.99 |
| *Spirochaetes* | 0.06±0.09% | 0.79±0.50% | 0.01 |
| *Actinobacteria* | 0.52±0.50% | 0.07±0.05% | 0.05 |
| *Euryarchaeota* | 0.01±0.01% | 0.17±0.15% | 0.03 |
| *Synergistetes* | 0.01±0.01% | 0.04±0.05% | 0.11 |
| *Candidatus Saccharibacteria* | 0.00±0.00% | 0.01±0.01% | 0.21 |
| *Planctomycetes* | 0.00±0.00% | 0.01±0.01% | 0.34 |
| *Deferribacteres* | 0.01±0.01% | 0.00±0.00% | 0.08 |
| *Verrucomicrobia* | 0.01±0.01% | 0.00±0.00% | 0.34 |
| *Lentisphaerae* | 0.01±0.01% | 0.00±0.00% | 0.34 |
| Other | - | - | - |

The relative abundance of phyla higher than 0.05% in two groups was shown. The predominant phyla (the average abundance of all samples higher than 1%) including *Bacteroidetes*, *Firmicutes*, *Proteobacteria*, *Fusobacteria* showed no significant differences between two groups. The proportion of *Spirochaetes* and *Euryarchaeota* in colon samples was significantly decreased in the weaned group (P < 0.05).

**Table S4 The family composition of colonic mi****crobiome in nursing and weaned piglets (n=6)**

| **tax_name** | **Weaned(n=6)** | **Nursing(n=6)** | **p** |
| --- | --- | --- | --- |
| *Prevotellaceae* | 20.44±13.83% | 37.12±4.68% | 0.02 |
| *Ruminococcaceae* | 19.48±16.81% | 19.98±9.44% | 0.95 |
| *Lachnospiraceae* | 20.28±13.30% | 7.43±2.33% | 0.04 |
| *Porphyromonadaceae* | 4.21±2.97% | 17.78±6.41% | 0 |
| *Bacteroidaceae* | 10.31±11.67% | 4.06±4.90% | 0.25 |
| *Lactobacillaceae* | 4.21±3.05% | 5.81±1.56% | 0.28 |
| *Fusobacteriaceae* | 4.40±6.81% | 4.63±7.35% | 0.96 |
| *Campylobacteraceae* | 6.7±10.23% | 0.32±0.32% | 0.14 |
| *Veillonellaceae* | 5.63±2.29% | 0.32±0.29% | 0 |
| *Acidaminococcaceae* | 0.94±0.58% | 0.73±0.25% | 0.44 |
| *Desulfovibrionaceae* | 0.29±0.28% | 1.06±0.48% | 0.01 |
| *Pasteurellaceae* | 0.10±0.12% | 1.15±1.47% | 0.11 |
| *Spirochaetaceae* | 0.06±0.09% | 0.92±0.59% | 0.01 |
| *Sutterellaceae* | 0.76±1.12% | 0.04±0.06% | 0.15 |
| *Rikenellaceae* | 0.03±0.04% | 0.77±0.53% | 0.01 |
| *Succinivibrionaceae* | 0.58±0.78% | 0.00±0.00% | 0.1 |
| *Coriobacteriaceae* | 0.53±0.51% | 0.05±0.04% | 0.05 |
| *Enterobacteriaceae* | 0.29±0.37% | 0.25±0.24% | 0.86 |
| *Methanobacteriaceae* | 0.01±0.01% | 0.19±0.18% | 0.03 |
| *Streptococcaceae* | 0.06±0.03% | 0.12±0.06% | 0.06 |
| Other | 0.31±0.36% | 0.29±0.12% | 0.89 |

The relative abundance of families higher than 0.05% in groups was shown. The most predominant families (the average abundance of all samples higher than 10%) were *Prevotellaceae* and *Ruminococcaceae*. Followed dominant families were *Lachnospiraceae*, *Porphyromonadaceae*, and *Bacteroidaceae*. *Ruminococcaceae* showed no significant differences between two groups (P >0.05). The proportion of *Prevotellaceae* and *Porphyromonadaceae* was significantly decreased in weaned group. And the relative abundance of *Lachnospiraceae* was significantly higher than that in nursing group (P < 0.05).

**Table S5 The genus composition of colonic microbiome in nursing and weaned piglets (n=6)**

| **tax_name** | **Weaned(n=6)** | **Nursing(n=6)** | **p** |
| --- | --- | --- | --- |
| *Prevotella* | 19.18±15.01% | 33.07±4.53% | 0.06 |
| *Bacteroides* | 11.53±12.53% | 5.22±4.53% | 0.28 |
| *Lactobacillus* | 5.76±4.37% | 8.86±3.31% | 0.2 |
| *Alloprevotella* | 1.81±2.23% | 12.81±4.99% | 0 |
| *Fusobacterium* | 4.72±7.25% | 5.43±8.41% | 0.88 |
| *Campylobacter* | 8.94±12.59% | 0.46±0.49% | 0.14 |
| *Faecalibacterium* | 9.02±9.06% | 0.05±0.04% | 0.04 |
| *Barnesiella* | 1.53±2.89% | 9.21±9.20% | 0.08 |
| *Roseburia* | 5.28±3.41% | 1.77±1.30% | 0.04 |
| *Oscillibacter* | 0.69±0.56% | 5.52±6.19% | 0.09 |
| *Clostridium XlVb* | 2.42±3.26% | 1.92±0.26% | 0.72 |
| *Megasphaera* | 3.86±4.45% | 0.08±0.11% | 0.06 |
| *Blautia* | 3.29±3.45% | 0.08±0.06% | 0.05 |
| *Clostridium XlVa* | 2.42±3.26% | 1.92±0.26% | 0.72 |
| *Flavonifractor* | 2.78±4.27% | 0.00±0.01% | 0.14 |
| *Gemmiger* | 2.10±2.56 | 0.61±0.64% | 0.2 |
| *Butyricicoccus* | 2.46±2.89% | 0.08±0.06% | 0.07 |
| *Parabacteroides* | 0.33±0.48% | 2.66±3.00% | 0.09 |
| *Phascolarctobacterium* | 1.24±0.77% | 1.12±0.54% | 0.28 |
| *Anaerovibrio* | 1.65±0.71% | 0.00±0.00% | 0 |
| Other | 9.47±2.68% | 10.15±2.73% | 0.67 |

The relative abundance of genera higher than 0.05% in two groups was shown. The most predominant genera (the average abundance of all samples higher than 10%) were *Prevotella* and *Bacteroides*, which showed no significant differences between two groups (P>0.05). Followed dominant genera were *Lactobacillus* and *Alloprevotella.* The relative abundance of *Alloprevotella* in Weaned group was significantly lower than that in the Nursing group in colonic microbiota (P < 0.05).

**
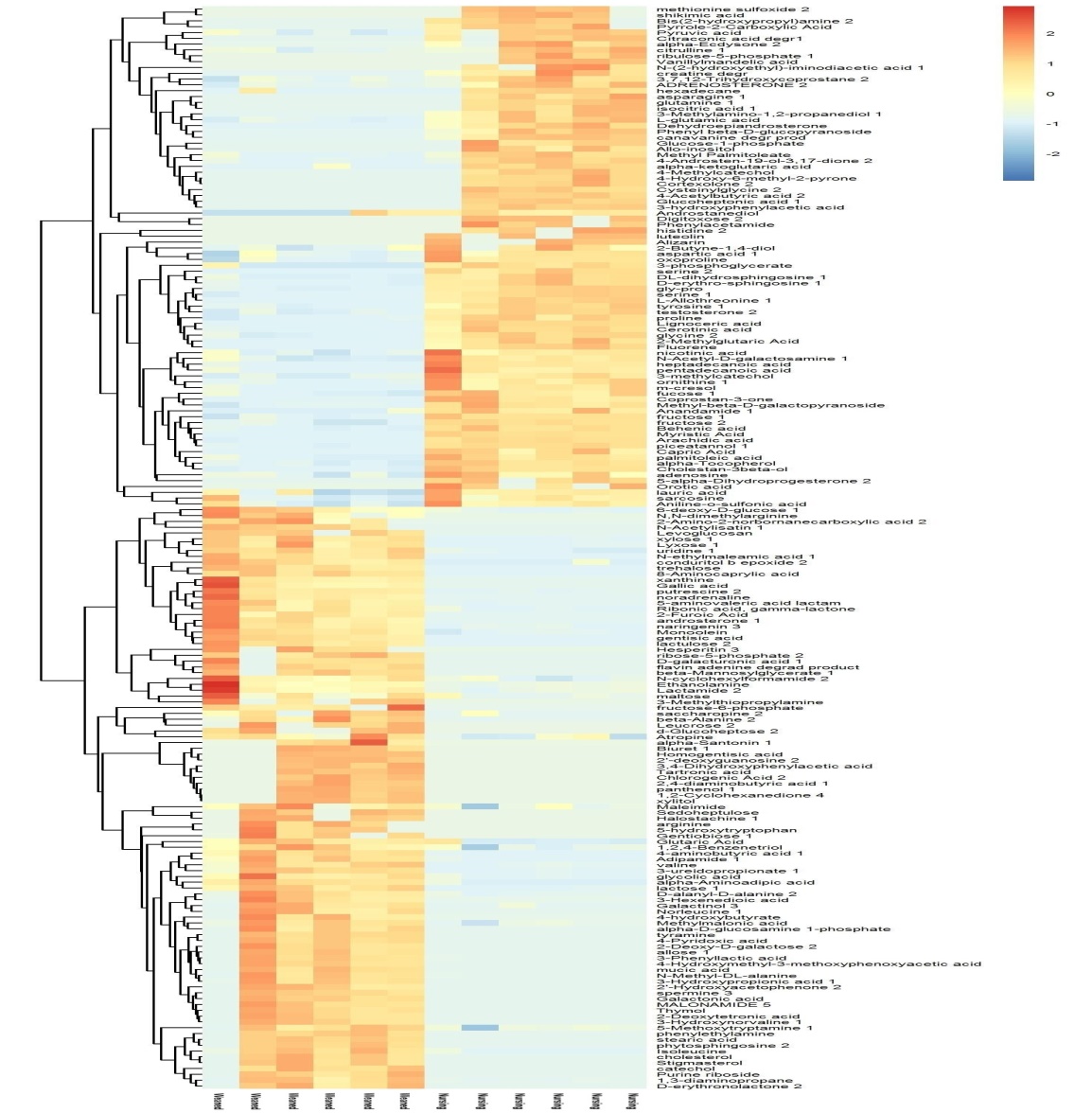
**

**Fig S1 Heatmap of hierarchical clustering analysis for Weaned and Nursing group.** The abscissa represents weaned and nursing groups, and the ordinate represents the differential metabolites between two groups. Color patches represent the relative expression of metabolites at the corresponding groups.
